# Uptake of intermittent preventive treatment for malaria in pregnancy among women in selected communities of Ebonyi State, Nigeria

**DOI:** 10.1186/s12884-019-2629-4

**Published:** 2019-12-02

**Authors:** Christian Obasi Akpa, Joshua Odunayo Akinyemi, Chukwuma David Umeokonkwo, Eniola Adetola Bamgboye, Tukur Dahiru, Ayo Stephen Adebowale, IkeOluwapo Oyeneye Ajayi

**Affiliations:** 1 Nigeria Field Epidemiology and Laboratory Training Programme, Abuja, Nigeria; 2 Department of Epidemiology and Medical Statistics, Faculty of Public Health, College of Medicine, University of Ibadan, Ibadan, Nigeria; 3 Department of Community Medicine, Alex Ekwueme Federal University Teaching Hospital, Abakaliki, Ebonyi State Nigeria; 4 Department of Community Medicine, Ahmadu Bello University, Zaria, Kaduna State Nigeria

**Keywords:** Malaria, Intermittent preventive treatment, Sulfadoxine/Pyrimethamine, Uptake, Nigeria

## Abstract

**Background:**

Malaria in pregnancy has adverse effects on maternal and child health. Intermittent preventive treatment (IPTp) with three doses of Sulfadoxine/Pyrimethamine is an effective preventive measure for malaria in pregnancy. However, 24.0% of women use this prophylactic regimen in Ebonyi State. Previous studies have focused on the level of uptake with less attention given to factors influencing uptake. Therefore, we examined the predictors of IPTp uptake in the last pregnancy among women in Ebonyi State, Nigeria.

**Methods:**

This was a community-based cross-sectional study among 340 women of reproductive age selected using multistage sampling technique. A semi-structured interviewer administered questionnaire was used to collect data on socio-demographic characteristics of respondents, IPTp uptake and reasons for not taking IPTp. Adherence was judged adequate if three or more doses of IPTp were taken, otherwise inadequate. Data were analyzed using descriptive statistics, Chi- square test and logistic regression model at 5% level of significance.

**Results:**

Mean age of respondents was 28.8 ± 5.2 years, 96.5% were married, 19.4% had tertiary education, and 11.2% were from polygamous family. Uptake of IPTp was 74.2%. The level of IPTp uptake was 12.5 and 41.0% among women with no formal and tertiary education respectively. A similar pattern of IPTp uptake was observed among women from monogamous (38.0%) and polygamous (39.5%) families. Women education, husband education and family type were associated with uptake of IPTp, however only husband education remained a predictor of uptake. Women whose husband had secondary education (aOR = 4.1, 95%CI: 1.66–10.06) and tertiary education (aOR = 4.8, 95%CI: 1.76–12.90) were more likely to have IPTp uptake than those whose husbands had below secondary education.

**Conclusion:**

Adequate IPTp uptake among women in their last pregnancy was below WHO recommendation. Intervention aimed at improving couple’s education could facilitate increase in IPTp uptake in Ebonyi State.

## Background

Malaria in pregnancy has consequences for mother, fetus and newborn child which are largely preventable [1]. World Malaria Report 2018 reaffirms that, the realization of the two critical 2020 milestones of the WHO Global Technical Strategy for malaria to reduce case incidence and death rates by at least 40% from 2015 levels, might not be feasible [2]. In 2018, there were an estimated 219 million cases and 435,000 deaths from malaria globally and about 80% of these deaths were found in the WHO African Region and India [2]. Only seven countries accounted for 53% of all global malaria death of which Nigeria contributed 19% [2].

Twenty-five million pregnant women are currently at risk of malaria, it accounts for over 10,000 maternal and 200,000 neonatal deaths per year in sub-Saharan Africa [3]. Inadequate access and uptake of lifesaving malaria interventions which includes intermittent preventive treatment in pregnancy (IPTp) has been reported as a predisposing factor to maternal and infant morbidity and mortality [2].

In areas of stable malaria transmission in Africa, WHO recommends a package of intermittent preventive treatment (IPTp) with Sulfadoxine–Pyrimethamine (SP) and use of insecticide-treated nets (ITNs), together with effective case management of clinical malaria and anaemia to prevent morbidity and mortality due to malaria in pregnancy [4]. A minimum of three doses of SP, taken one month apart, commencing after quickening (approximately 18 weeks gestation) and routinely delivered at antenatal clinics has been recommended [4]. Among 33 African countries that reported IPTp coverage in 2018, 22% of eligible pregnant women received the recommended three or more doses of IPTp, compared with 17% in 2015 and 0% in 2010 [2].

Nigeria adopted the IPTp strategy in year 2005 [5]. However, variation has been found in the level of IPTp uptake across the states in Nigeria. According to Nigeria Demographic and Health Survey 2018, the percentage of women who received at least one dose of SP was 63.6%, (72.6% urban and rural 58%) but the proportion of women who took at least three doses of SP was reported as 16.6% (urban 20.7% and rural 14%). The proportion of women who took at least three doses in Ebonyi State was 24.2% [6]. A household survey on IPT coverage among 1307 respondents that compared urban and rural areas of Enugu, Nigeria in 2012, found that uptake remained low at 13.7% [7]. Whereas, more parasitemia had been reported among pregnant women who did not received IPTp in Ebonyi State, there appears to be paucity of studies documenting the uptake levels of IPTp and exploring the factors associated with uptake [8]. We determined the level of IPTp uptake and factors influencing it among women of reproductive age residing in communities in Ebonyi State.

## Methods

### Study area

This study was conducted in Ebonyi State which is one the five states that make up the South East geopolitical zone in Nigeria. Ebonyi State has 13 Local Government Areas (LGA) and 142 communities. The 2019 projected population of Ebonyi State and population of women of reproductive age were 3,112,220 and 684,688 respectively as obtained from the State Ministry of Health. Presently there are 556 health facilities both public and private in Ebonyi State comprising one tertiary health facility, 13 general hospitals, 6 mission hospitals, 417 primary health centers and 119 private hospitals/clinics [9]. Most of these health facilities offer maternal and child health related services such as ante natal care (ANC) and immunization services. The ANC attendance at health facilities with skilled providers was 70.3% [6]. Ebonyi State is one of the states supported by the Presidential Malaria Initiative project. The project provides training for health workers, provides malaria preventive commodities including SP. During ANC clinics, SP is prescribed and dispensed at the health facility at no cost to the pregnant.

The predominant occupations in the state are farming and trading. Most communities in Ebonyi State engage in farming activities which increases the man-vector contact aiding malaria transmission. More so, the limestone excavations and quarrying activities in many of the communities create enabling environments for water accumulation and breeding of mosquitoes that transmit malaria.

### Study design and population

We conducted a cross-sectional study among women of reproductive age (*N* = 340). We included women who had been residing in selected communities and had given birth in the past year prior to the survey. The women who were incapacitated or not disposed to respond to the interview were excluded.

### Sampling technique

Multistage sampling technique was used to recruit respondents for the study. Two LGAs (Abakaliki and Ebonyi) were selected by balloting from a list of 13 LGAs in the state. In each of the LGAs, four communities were selected by balloting from Abakaliki LGA (Izziunuhu, Azuiyiokwu, Okpaugwu, and Timber Communities) and Ebonyi LGA (Aboffia, Kpirikpiri, Abakpa, and New Layout). The list of households in these communities were obtained from the immunization department of the selected LGAs. A total of 1041 households from the eight communities were obtained to serve as sampling frame. Eligible women were selected from each of the sampled households in the communities using systematic sampling technique until the allocated sample size was achieved. However, in any household where there was more than one eligible woman, the respondent was selected by balloting.

### Measurement of variables

The dependent variable was IPTp uptake which was categorized based on the number of doses of IPTp they took during the pregnancy in the year preceding the survey. Those who did not take any IPTp were categorized as none, those who took less than three doses were categorized as inadequate uptake and those who took three doses and or more were categorized as having adequate uptake. To examine the determinants of IPTp uptake, we re-categorize the variable into two groups. None IPTp uptake and inadequate uptake were merged and categorized as inadequate while adequate uptake remained as ≥3 doses.

### Data collection and analysis

A semi-structured interviewer administered questionnaire was used to collect data on socio-demographic characteristics of respondents, uptake of intermittent preventive treatment for malaria in pregnancy, health facility type and spouse information. The data collection was conducted by trained research assistants. Data were analyzed with Statistical Package for Social Science (IBM SPSS) version 20. We examined the association between uptake of IPTp and sociodemographic characteristics using Chi Squared test. The variables that were significant at 10% in the bivariate analysis were included in the multiple logistic regression. We identified the predictors of IPTp uptake at 5% level of significance.

### Ethical considerations

We obtained ethical approval from the Research and Ethics Committee of Ebonyi State Ministry of Health (approval reference number: SMOH/ERC/054/19). Written informed consents were obtained from all respondents after explaining the details of the study. Participation in the study was voluntary and the information obtained were handled with strict confidentiality.

## Results

The mean age of the respondents was 28.8 ± 5.2 years. The 25–34 years age group constituted the majority (65.0%) and most of the respondents were married 328 (96.5%). Majority of the respondents (67.9%) and their spouses (55.0%) had attained secondary level education (Table 1).

**Table 1:** Sociodemographic and reproductive characteristics of the respondents (*N* = 340)

Variable	Frequency	Percentage
Age (years)		
< 25	69	20.3
25–34	221	65.0
≥ 35	50	14.7
Marital status		
Married	328	96.5
Not married	12	3.5
Employment status		
Employed	301	88.5
Unemployed	39	11.5
Respondents’ education		
No formal	8	2.4
Primary	35	10.3
Secondary	231	67.9
Tertiary	66	19.4
Husband education		
No formal	9	2.6
Primary	43	12.7
Secondary	187	55.0
Tertiary	101	29.7
Family type		
Monogamous	292	85.9
Polygamous	38	11.2
Single mother	10	2.9
Parity		
Primigravida	51	15.0
Multigravida	220	64.7
Grand multipara	69	20.3

Two hundred and forty-five women (74.2%) took at least one dose of IPTp during the last pregnancy within a year prior to the study, whereas, 130 (53.0%) took at least three doses (Table 2).

**Table 2:** Uptake of intermittent preventive treatment (IPTp) among respondents

Variable	Frequency	Percentage
Took antimalaria in pregnancy in the year preceding the survey		
Yes	330	97.1
No	10	2.9
Type of antimalaria used (*n* = 330)		
Sulfadoxine/Pyrimethamine (SP)	245	74.2
ACT/ Coartem	24	7.3
Chloroquine	8	2.4
Quinine	2	0.6
I don’t know	34	10.3
I can’t remember	17	5.2
Number of times SP was taken (*n* = 245)		
Once	21	8.6
Twice	94	38.4
Three times	113	46.1
More than three times	17	6.9
SP was taken at health facility (during ANC, *n* = 245)		
Yes	240	98.0
No	5	2.0
Type of health facility SP was received (*n* = 240)		
Tertiary government owned	40	16.7
Private mission owned	112	46.7
Primary health centre	50	20.8
Private for-profit hospitals	38	15.8

**Fig. 1:**
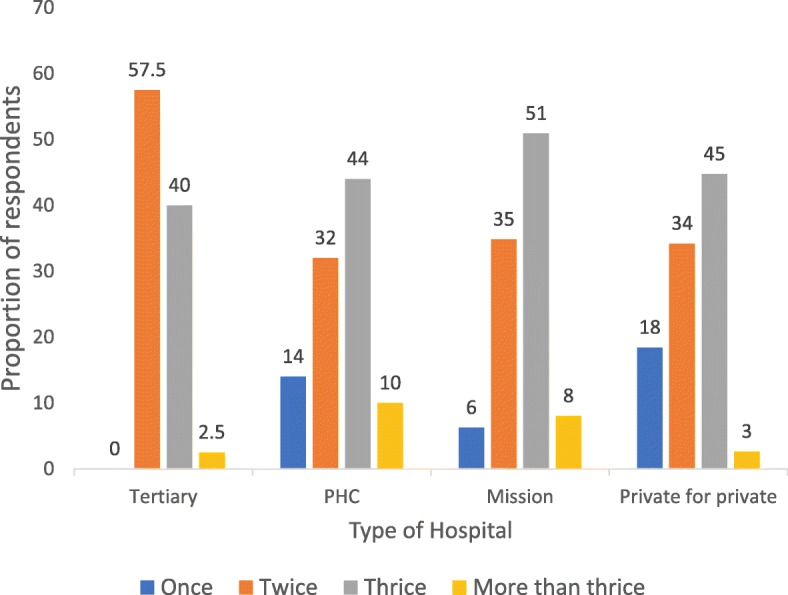
Distribution of IPTp uptake by doses and Health Facility type

Out of 40 respondents receiving ANC from tertiary health facility, 57.5% did not receive up to three recommended IPTp during pregnancy compared to those attending ANC in the other health facility types. Up to half of the those attending ANC in mission owned health facilities (51%) received at least three doses of IPTp during pregnancy (Fig. 1).

Respondent’s education (*p* = 0.002), the spouse level of education (*p* < 0.001) and family type (p = 0.002) were found to be significantly associated with uptake of IPTp among the respondents at bivariate analysis (Table 3). However, when these were modelled in binary logistic regression, only husband education predicted uptake of IPTp. Respondents whose husbands had attained secondary education were 4 times more likely to take IPTp (adjusted odds ratio [aOR]:4.1; 95% Confidence interval [CI]: 1.64–10.06) compared to those whose husbands had attained primary education or less. Similarly, respondents whose husbands had attained tertiary education were 4.8 times more likely to take IPTp (aOR:4.8; 95%CI: 1.76–12.90) compared to those whose husbands had attained primary education or less (Table 4).

**Table 3:** Association between socio-demographic characteristics of the respondents and level of IPTp uptake

Variable	Uptake of IPTp			Chi square (χ^2^)	*p* value
	None	Inadequate (1 or 2 doses)	Adequate (≥ 3 doses)		
Age (years)					
< 25	17 (24.6)	31 (44.9)	21 (30.4)	6.407	0.171
25–34	61 (27.6)	72 (32.6)	88 (39.8)		
≥ 35	17 (34.0)	12 (24.0)	21 (42.0)		
Marital status					
Married	95 (29.0)	109 (33.2)	124 (37.8)	4.888	0.087
Not currently married	0 (0.0)	6 (50.0)	6 (50.0)		
Respondent education					
No formal	3 (37.5)	4 (50.0)	1 (12.5)	21.144	0.002
Primary	20 (57.1)	7 (20.0)	8 (22.9)		
Secondary	60 (26.0)	77 (33.3)	94 (40.7)		
Tertiary	12 (18.2)	27 (41.0)	27 (41.0)		
Husband education					
No formal	6 (66.7)	3 (33.3)	0 (0.0)	28.431	< 0.001
Primary	23 (53.5)	13 (30.2)	7 (16.3)		
Secondary	45 (24.1)	64 (34.2)	78 (41.7)		
Tertiary	21 (20.8)	35 (34.7)	45 (44.6)		
Family type					
Monogamous	92 (31.5)	89 (30.5)	111 (38.0)	16.581	0.002
Polygamous	3 (7.9)	20 (52.6)	15 (39.5)		
Single mothers	0 (0.0)	6 (60.0)	4 (40.0)

**Table 4:** Predictors of adequate uptake of IPTp among the respondents

Variable	Crude OR	Adjusted OR
Age (years)		
< 25	1	
25–34	1.5 (0.93–2.46)	
≥ 35	1.7 (0.87–3.13)	
Respondent education		
Primary or less	1	
Secondary	2.6 (1.1–5.66)	1.4 (0.58–3.32)
Tertiary	2.6 (1.1–6.33)	1.3 (0.46–3.63)
Family type		
Monogamous	1	
Polygamous	1.1 (0.60–1.90)	1.2 (0.58–2.47)
Single mother	1.1 (0.37–3.20)	0.8 (0.21–3.07)
Husband education		
Primary or less	1	
Secondary	4.6 (2.26–9.37)	4.1 (1.64–10.06)
Tertiary	5.2 (2.45–10.88)	4.8 (1.76–12.90)
Parity		
Primigravida	0.9 (0.46–1.72)	
Multigravida	1.6 (0.97–2.53)	
Grand multipara	1	

*inadequate = (none and < 3 doses of IPTp), Adequate = ≥3 doses

## Discussions

This study was on uptake of intermittent preventive treatment for malaria in pregnancy among women who had given birth in the past year prior to the survey, in selected communities in Ebonyi State. We found a relatively high uptake of at least one dose of IPTp among the respondents. This could be due to growing level of awareness of IPTp in the country. The finding in our study was higher than that reported in Enugu in 2012 among 1307 respondents [7]. It was also higher than the south east regional average uptake found in the 2015 Nigeria Malaria Indicator Survey which was conducted among 1338 respondents in each geo-political zone [10]. The uptake in our study was slightly higher than the 67.2% reported for Ebonyi State in the 2018 Nigeria Demographic Health Survey [6]. It was also higher than other studies in South West Nigeria [11] and Mali [12] but lower than that reported in Ghana [13]. The higher uptake observed among the primary health facilities compared to the private and tertiary health facilities could be related to the practice observed in the state. Women tend to register for ANC early in facilities closer to them due to cost of services and transportation but register at the private or tertiary health facilities close to their delivery. The late registration at this hospital types could have accounted for the apparent lower level of uptake. The relationship between time of first ANC attendance and uptake of IPTp has been reported. This however needs more exploration in our environment.

A 2018 survey that was done in eight sub-Saharan African countries among 18,603 respondents reported a low uptake of IPTp at 25.9% [14]. The level of uptake in our study implies that the uptake of IPTp is improving and that pregnant women in Ebonyi State may likely have a better health seeking behavior towards prevention of malaria in pregnancy with the use of IPTp. It is important to note that Ebonyi State is one of the states supported by Presidential Malaria Initiative in Nigeria. The high prevalence may be due to the effect of the program activities on malaria control in the state. This finding could also be due to the nature of the study setting which were largely urban and the high level of education of the respondents. The proportion of women who had adequate uptake of IPTp (three or more doses) observed in our study was high but the WHO recommended target of 100% is yet to be achieved. The proportion of women who had adequate uptake was higher than that reported in a metropolitan area in Ghana [13].

The woman’s level of education, husband’s level of education and family type were associated with the uptake of IPTp. However, only husband’s level of education was a predictor of adequate uptake of IPTp among respondents. This could be due to the influence of education on knowledge of IPTp and its benefits. The finding supports the importance of male involvement in antenatal care services. Male education is an important factor that could help improve antenatal care services use and IPTp uptake by pregnant women. Having secondary or tertiary education improved the odds of the uptake of IPTp by pregnant women by four folds. Contrary to our finding, Nigeria malaria indicator survey 2015 found that woman’s education was a predictor of IPTp uptake [10]. A cross-sectional study conducted in south west Nigeria in 2012, and a qualitative study that was done in Uganda showed that lack of knowledge on the benefit of IPTp constituted a barrier to its uptake [15]^,^[16]. There is a tendency for the more educated to have more knowledge on IPTp, have access to health services, better risk perception, better income and adhere to recommendation. A cross sectional study in Kano, North West Nigeria in 2012 among 239 women found that advanced maternal age and higher level of education were predictors of IPTp uptake [17]. Maternal education did not however predict uptake in our study.

It is important to note that high proportion of pregnant women took their SP at the health facility under direct observation of the health worker. This could have contributed to the high level of uptake observed. This also suggests availability of SP at the health facility which could have encouraged the uptake. It has also been reported that early initiation of ANC was associated with good uptake of SP [13].

A limitation of this study was the possibility of a recall bias. To minimize this, the respondents were shown pictures of Sulfadoxine/Pyrimethamine package to enable them identify the medicine and correctly recall taking it. In addition, the study included only women who had given birth in the past year prior to the survey to aid their recall hence may not be representative of the target population. Information on the gestational age at first ANC visit and the number of ANC visits were not captured in this study. This could have enabled more exploration on the factors that influenced uptake of the IPTp.

## Conclusion

Adequate IPTp uptake among women in their last pregnancy was below WHO recommendation. Age, family type, respondent’s education, parity and husband education were associated with uptake of IPTp among pregnant women. However, husband education remained a determinant of IPTp uptake among the women in this study. We recommend that male education should be encouraged. This would probably have a positive influence on uptake of IPTp.

## Data Availability

The datasets used and/or analysed during the current study are available from the corresponding author on reasonable request.

## Abbreviations

ANC: Antenatal Care
ERC: Ethics and Research Committee
IPTp: Intermittent Prophylactic Treatment in Pregnancy
ITN: Insecticide Treated Nets
SMOH: State Ministry of Health
SP: Sulfadoxine/Pyrimethamine
WHO: World Health Organization
